# Alzheimer’s disease induced neurons bearing *PSEN1* mutations exhibit reduced excitability

**DOI:** 10.3389/fncel.2024.1406970

**Published:** 2024-10-09

**Authors:** Simon Maksour, Rocio K. Finol-Urdaneta, Amy J. Hulme, Mauricio e Castro Cabral-da-Silva, Helena Targa Dias Anastacio, Rachelle Balez, Tracey Berg, Calista Turner, Sonia Sanz Muñoz, Martin Engel, Predrag Kalajdzic, Leszek Lisowski, Kuldip Sidhu, Perminder S. Sachdev, Mirella Dottori, Lezanne Ooi

**Affiliations:** ^1^School of Chemistry and Molecular Bioscience and Molecular Horizons, University of Wollongong, Wollongong, NSW, Australia; ^2^School of Medical and Indigenous Health Science and Molecular Horizons, University of Wollongong, Wollongong, NSW, Australia; ^3^Translational Vectorology Research Unit, Children’s Medical Research Institute, Faculty of Medicine and Health, The University of Sydney, Westmead, NSW, Australia; ^4^Australian Genome Therapeutics Centre, Children’s Medical Research Institute and Sydney Children’s Hospitals Network, Westmead, NSW, Australia; ^5^Laboratory of Molecular Oncology and Innovative Therapies, Military Institute of Medicine – National Research Institute, Warsaw, Poland; ^6^Centre for Healthy Brain Ageing, School of Clinical Medicine, University of New South Wales, Sydney, NSW, Australia

**Keywords:** Alzheimer’s disease, *PSEN1*, neuronal excitability, iNs, iPSCs

## Abstract

Alzheimer’s disease (AD) is a devastating neurodegenerative condition that affects memory and cognition, characterized by neuronal loss and currently lacking a cure. Mutations in *PSEN1* (Presenilin 1) are among the most common causes of early-onset familial AD (fAD). While changes in neuronal excitability are believed to be early indicators of AD progression, the link between *PSEN1* mutations and neuronal excitability remains to be fully elucidated. This study examined iPSC-derived neurons (iNs) from fAD patients with *PSEN1* mutations S290C or A246E, alongside CRISPR-corrected isogenic cell lines, to investigate early changes in excitability. Electrophysiological profiling revealed reduced excitability in both *PSEN1* mutant iNs compared to their isogenic controls. Neurons bearing S290C and A246E mutations exhibited divergent passive membrane properties compared to isogenic controls, suggesting distinct effects of *PSEN1* mutations on neuronal excitability. Additionally, both *PSEN1* backgrounds exhibited higher current density of voltage-gated potassium (Kv) channels relative to their isogenic iNs, while displaying comparable voltage-gated sodium (Nav) channel current density. This suggests that the Nav/Kv imbalance contributes to impaired neuronal firing in fAD iNs. Deciphering these early cellular and molecular changes in AD is crucial for understanding disease pathogenesis.

## 1 Introduction

Alzheimer’s disease (AD) is a devastating, progressive neurodegenerative disease that affects memory and cognition and is characterized by the loss of neurons. Thus, an important area of research involves examining how early molecular changes may influence the cause and progression of AD. These studies focus on early changes in gene expression (Guennewig et al., 2021), energy metabolism (Johnson et al., 2020), altered neurogenesis and neuronal differentiation (Arber et al., 2021; Meyer et al., 2019), and neuronal firing (Ghatak et al., 2019; Spoleti et al., 2022). Multiple lines of evidence suggest that changes in neuronal excitability are an early phenotype of neurodegeneration that may drive disease pathology in neurodegenerative diseases, including AD (as reviewed in Targa Dias Anastacio et al., 2022) and amyotrophic lateral sclerosis (as reviewed in Do-Ha et al., 2018). Neuronal excitability changes are influenced by the two major hallmarks of AD, Aβ (Busche et al., 2012; Busche et al., 2008) and tau pathology (Busche et al., 2019; Crimins et al., 2012), whilst conversely, excitability changes have also been shown to drive both the deposition of both of these AD hallmark pathologies (Cirrito et al., 2005; Pooler et al., 2013; Wu et al., 2016; Yamamoto et al., 2015). Whilst excitatory neurons demonstrate excitability changes, there are also demonstrated contributions from inhibitory neurons (Ghatak et al., 2019; Nuriel et al., 2017; Verret et al., 2012) and glial cells (Targa Dias Anastacio et al., 2022). Furthermore, correcting neuronal activity through pharmacological or genetic intervention in AD mouse models improves memory and cognition (Martinez-Losa et al., 2018; Ping et al., 2015; Roberson et al., 2007), highlighting the role of neuronal excitability regulation in disease progression.

*PSEN1* is the most common causative gene for early-onset, familial AD (fAD) and is believed to contribute to neuronal vulnerability through the overproduction of amyloid-ß (Aβ) peptides, which results in the generation of Aß plaques in the brain (Ooi et al., 2020). There are over 300 known mutations in *PSEN1*, many with pathogenic outcomes, however the effects of each mutation on the disease phenotype remains to be fully elucidated (*PSEN1* mutations database, ALZforum).^1^ The implications of *PSEN1* mutations on neuronal vulnerability have been assessed in animal models and human cell models of disease, including patient induced pluripotent stem cell (iPSC) derived neurons. In addition to disrupted amyloid precursor protein (APP) processing and plaque formation, *PSEN1* mutations induce early changes in neurons, including, increased susceptibility to Aβ (Armijo et al., 2017) and ferroptosis (Greenough et al., 2022), dysregulated neurogenesis and differentiation (Arber et al., 2021; Hurley et al., 2023; Vanova et al., 2023), decreased neurite outgrowth (Balez et al., 2016; Dowjat et al., 1999; Furukawa et al., 1998; Ghatak et al., 2019), endosomal dysfunction (Kwart et al., 2019) and alterations in neuronal excitability (Chen et al., 2021; Hurley et al., 2023; Vitale et al., 2021). These studies highlight the complex role of presenilin-1 in multiple cellular functions, and the need to understand how specific mutations affect neuronal processes.

Human iPSCs offer an avenue to generate neurons from patients bearing disease-relevant gene mutations, and interrogate the intrinsic differences in excitability properties of neurons in the absence of late-stage AD pathology and supporting cell types. Therefore, this study aimed to use iPSC-derived neurons from two AD patients bearing pathogenic mutations in *PSEN1*, S290C or A246E, along with their CRISPR-corrected isogenic controls to investigate early neuronal excitability changes in disease. The S290C mutation, which results in the deletion of exon 9, was originally identified in a Finnish family and displayed typical Aß plaques, neurofibrillary tangles (NFTs) and gliosis, in addition to “cotton wool” plaques and hippocampal atrophy (Crook et al., 1998; Verkkoniemi et al., 2000). The A246E mutation was originally identified in a Canadian family, with 52 affected family members spanning eight generations, with *post mortem* tissue highlighting atrophy of the frontal lobe and hippocampus, neuronal loss, Aß plaques and NFTs (Nee et al., 1983). At a neuronal level, the A246E mutation alters tissue specification in organoids (Vanova et al., 2023), increased susceptibility to Aß (Armijo et al., 2017), increased vulnerability to ferroptosis (Greenough et al., 2022) and altered Ca^2+^, glutamate and NMDA signaling (Balez et al., 2024; Targa Dias Anastacio et al., 2024). The S290C mutation also altered AMPA signaling (Targa Dias Anastacio et al., 2024). Furthermore, the deletion of exon 9 in neurons increased vulnerability to ferroptosis (Greenough et al., 2022) and impaired endocytosis (Woodruff et al., 2016). It is currently unknown whether these mutations cause differences in intrinsic firing properties in excitatory neurons. Understanding the early common and divergent cellular and molecular changes disrupted in neurons from AD patients bearing different *PSEN1* mutations will provide insight into the functional impact of disease-causing mutations and shed light on the mechanisms underlying the specific vulnerability of excitatory neurons.

## 2 Methods

### 2.1 Cell culture

#### 2.1.1 iPSC cell lines and maintenance

Use of iPSC lines for this project was approved by the UOW Human Ethics Committee (#2017–375, 2017–382, 2020–450, 2020–451, 13–299). This study used iPSCs generated from early-onset AD patients with a *PSEN1* S290C (S290C) (Supplementary Figure S1) or a A246E mutation (A246E) (Muñoz et al., 2018) and their respective CRISPR-corrected isogenic controls, S290^IC^ (Supplementary Figure S1) and A246^IC^ (Targa Dias Anastacio et al., 2024, in revision). The iPSCs were cultured as previously reported in Abu-Bonsrah et al. (2019), Denham and Dottori (2011), and Mattei et al. (2019). Briefly, the iPSCs were maintained in mTesR1 (StemCell Technologies, #85850) on matrigel-coated tissue culture ware, kept in normoxic conditions at 37°C with 5% CO_2_. Cells were passaged once every 5–7 days using 0.5 mM EDTA (Life Technologies, #AM9260G) in PBS^−/−^ (Life Technologies, #14190250). Methods on the cell line generation and characterization can be found in the Supplementary materials.

### 2.2 Lentiviral production

Viral particles containing an open reading frame of *Neurogenin-2* (*NGN2*) were produced to differentiate iPSCs into mature neurons as described in Hulme et al. (2020) and Maksour et al. (2024). Briefly, HEK293T cells were transfected with the DNA of lentiviral packaging plasmids vSVG (Addgene, USA, #8454), RSV (Addgene, #12253), pMDL (Addgene, #12251), and either the tetracycline transactivator (TTA) vector, M2rtTA (Addgene, #20342), or the *NGN2* overexpression vector, TetO-*NGN2*-eGFP-Puro plasmid (Addgene, #79823) using Polyethyleneimine (Sigma-Aldrich, USA, #408727). DNA was added in a ratio of 4:2:1:1, transfer vector:pMDL:RSV:vSVG. The cell culture media containing viral particles was collected every 24 h over 3 days. The viral supernatant was concentrated 200× by ultracentrifugation at 66,000 × *g* for 2 h at 4°C. The viral pellet was resuspended in PBS and stored at −80°C until needed.

### 2.3 Generation of NGN2-induced neurons (iNs)

This study used a protocol published in Maksour et al. (2024) to generate mature neurons via NGN2 overexpression. Briefly, iPSCs were resuspended as single cells using Accutase for 2–3 min at RT. Single cells were plated at 15,000 cells/cm^2^ onto 10 μg/mL poly-D-lysine (PDL) and laminin (LAM) coated culture plate in mTeSR1 media supplemented with 10 μM Y27632. Cells were allowed to attach for 6–8 h, after which 0.5 μL of viral particles of both NGN2 overexpression and the TTA per 15,000 cells. Virus was removed 16–20 h following transduction with fresh neural media [Neurobasal medium (NBM; Life Technologies, #21103–049) supplemented with 1× N-2 supplement 1× B-27 supplement, 1× Insulin-Transferrin-Selenium-A and 2 mM L-glutamine] supplemented with 1 μg/mL doxycycline (DOX; Sigma-Aldrich, # D9891), 10 μM SB431542 and 0.1 μM LDN193189 to promote a cortical fate. After 24 h of DOX induction, 0.5 μg/mL puromycin was added daily for 3 days for selection of successfully transduced cells, in addition to DOX, SB431542 and LDN193189. Following selection fresh media supplemented with 10 μg/mL BDNF was added. Following selection, BrainPhys media [Brainphys medium (StemCell Technologies, #05790) supplemented with NeuroCult SM1 (without vitamin A; StemCell Technologies, #05731) and N2 supplement-A (StemCell Technologies, #07152)] was subsequently added in at increasing concentrations (25–100% in NM) for each media change to improve maturation. Neurons were assessed by whole-cell patch clamp between 21 and 35 days post viral transduction.

### 2.4 Electrophysiology

Sterile plastic coverslips, cut into 10 mm slides, were coated with PDL and LAM before plating NGN2 iNs for functional characterization. Whole-cell patch clamp recordings were performed on matured neurons aged 3–5 weeks, following the protocol outlined in Hulme et al. (2020). Recordings were conducted at room temperature (20–22°C) using a MultiClamp 700B Amplifier, digitized with a Digidata 1,440, and controlled via pClamp11 software (Molecular Devices). Whole-cell membrane currents were measured at 100 kHz, with series resistance compensated at 60–80%. Fire-polished borosilicate patch pipettes with a resistance of 2–4 MΩ were employed with an intracellular buffer composed of (in mM) 140 K-gluconate, 10 NaCl, 2 MgCl_2_, 10 HEPES, 5 EGTA (pH 7.2, osmolality 295 ± 5 mOsm/kg). The bath solution for current clamp experiments contained (in mM) 135 NaCl, 2 CaCl_2_, 2 MgCl_2_, 5 KCl, 10 glucose, 10 HEPES (pH 7.4, osmolality 315 ± 5 mOsm/kg).

Under current-clamp conditions, the resting membrane potential (RMP) was measured as the average membrane voltage without any current injection. Total membrane capacitance was estimated by automatically integrating the transient capacitive current produced during voltage-clamp steps, using pClamp11 (Molecular Devices). To analyze the intrinsic properties and excitability of iNs, 1-s current steps ranging from −150 to 140 pA in 10-pA increments were injected, starting from the RMP. Input resistance (Rin) was calculated from the steady-state voltage responses to 1-s hyperpolarizing currents, using the slope of the voltage–current relationship. Hyperpolarizing current induced membrane potential change (ΔMP) was defined as the difference between the minimum membrane potential recorded during the −100 pA current injection and the RMP. Rheobase was determined as the minimal current required to trigger an action potential (AP). AP characteristics were analyzed using Clampfit 11 (Molecular Devices). The AP threshold was defined as the voltage at which dV/dt exceeded 10 mV/ms, and AP amplitude was measured as the difference between the threshold and peak voltage. AP half-width was calculated as the duration at 50% of the AP amplitude. The threshold potential relative to the baseline was estimated by identifying the intersection of the AP’s rising phase and the slope leading up to its initiation, using a three-point tangent slope vector to find where the slope reached or exceeded 10 mV/ms. Under voltage-clamp conditions, total voltage-dependent sodium (INav) and potassium (IKv) currents were quantified at their peak amplitudes. INav was measured at the minimum current inflection at −10 mV, and IKv at the maximum current observed at 20 mV. These current values were then normalized by dividing by the capacitance of the respective iNs and reported as conductances in pA/pF.

### 2.5 Statistical analysis

Experiments were performed with 3–4 independent experimental differentiations (*n* = 3–4, biological replicates). Statistical analyses were conducted using GraphPad Prism software, version 10 (GraphPad Software, La Jolla, USA). Data was determined to be normally distributed using a Shapiro–Wilk normality test, where data were normally distributed, data were analyzed by a multiple t-test with Holm-Sidak method, unless stated otherwise. An *α* of 0.05 (*p*-value <0.05) was considered statistically significant.

## 3 Results

### 3.1 The iPSC derived neurons from two different fAD *PSEN1* backgrounds exhibit impaired excitability

**Figure 1:**
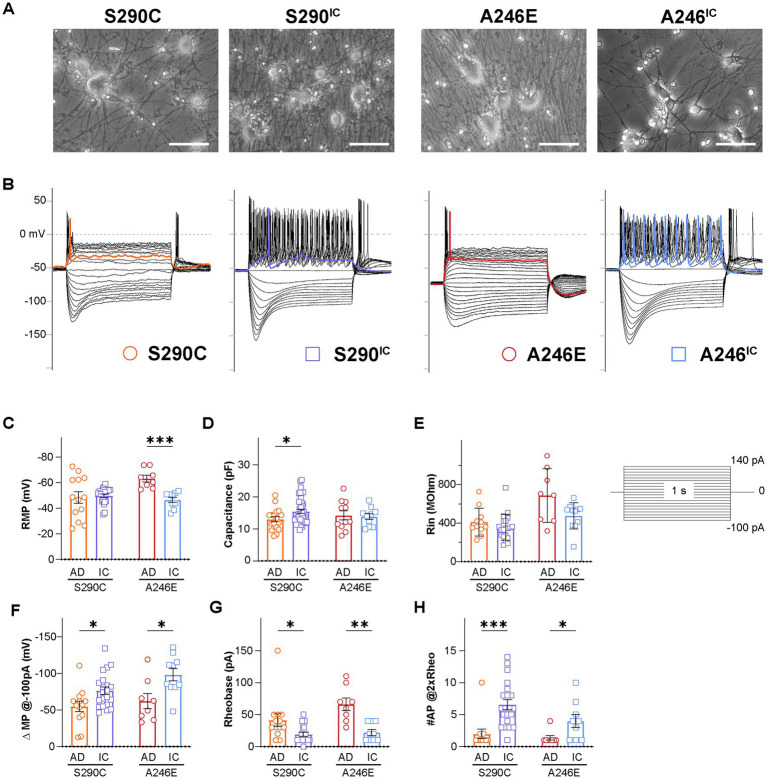
Neurons derived from AD patients bearing a *PSEN1* mutation exhibit reduced excitability. Whole cell patch clamping was performed on day 21–35 NGN2 iNs derived from fAD patients with a *PSEN1*^S290C^ (S290C) and *PSEN1*^A246E^ (A246E) and their respective CRISPR-corrected isogenic controls, S290^IC^ and A246^IC^ **(A)**. **(B)** Representative current clamp recordings of membrane potential responses of all cell lines. The stimulation protocols consisted of one-second-long current injections from −100 pA to 140 pA in 10 pA steps. The colored traces indicate firing activity at rheobase. Neurons were tested for **(C)** resting membrane potential (RMP), **(D)** capacitance, **(E)** input resistance (Rin), **(F)** hyperpolarizing current induced membrane potential change (ΔMP), **(G)** rheobase, and **(H)** the number of action potentials fired at 2 times rheobase. Data is presented as the mean ± SEM. Each data point represents an individual cell (*n* = 7–41), from 3 independent differentiations. Data was analyzed using multiple t-tests with Holm-Sidak for multiple comparisons where *p < 0.05, **p < 0.01, ***p < 0.001, and ****p < 0.0001. AD, Alzheimer’s disease; IC, isogenic control.

We have previously demonstrated the forced overexpression of NGN2 successfully and robustly generates functional, glutamatergic excitatory induced neurons (iNs) (Maksour et al., 2024). This study generated iNs from iPSCs of fAD patients bearing the *PSEN1*^S290C^ (S290C) and *PSEN1*^A246E^ (A246E) and their respective CRISPR corrected counterparts, S290^IC^ and A246^IC^, to assess their electrophysiological properties (Figure 1A). Previous studies from our team have shown that iNs derived from patients with S290C and A246E mutations do not exhibit tau or Aβ pathology within 35 days in culture (Targa Dias Anastacio et al., 2024). This model allows us to investigate neuronal excitability alterations in a controlled environment without the confounding effects of disease pathology or the presence of glial cells. Therefore, it provides a unique opportunity to study the intrinsic effects of these mutations on neuronal ion channel function. Neurite analysis of fAD and isogenic corrected control iNs was performed using the Incucyte live-cell imager to assess neurite properties (Supplementary Figure S2). The neurite length and branch points of S290C iNs were significantly decreased compared to the respective controls, further supporting the observation of decreased capacitance and consistent with our previous findings in AD neurons (Balez et al., 2016). Neuronal excitability was evaluated by manual patch-clamp in the whole-cell configuration with representative recordings displayed in Figure 1B with data for all parameters summarized in Supplementary Table S2.

The resting membrane potential (RMP) was evaluated within 2 min of switching to the current-clamp mode. RMP showed no difference between S290C and S290^IC^ iNs, while A246E iNs had significantly lower RMP compared to their isogenic controls (−63.3 ± 2.7 mV, *n* = 8 vs. −46.6 ± 2.9 mV, *n* = 9, respectively; *p* < 0.001) (Figure 1C). S290C iNs had significantly lower capacitance than S290^IC^ (13.0 ± 0.8 mV, *n* = 17 vs. 15.5 ± 0.6 mV, *n* = 41, respectively, *p* < 0.05) reflecting the generation of smaller iNs from this fAD line (Figure 1D), while A246E and A246^IC^ capacitance values were comparable. The iNs were stimulated through to 1 s long step current injections ranging from −150 pA to 140 pA. A comparison of the slopes of the steady-state voltage responses hyperpolarizing currents (−150 pA to −20 pA) revealed no differences in the input resistance (Rin) between the two fAD *PSEN1* mutants and their corrected controls (S290C vs. S290^IC^ *p* = 0.2397; A246E vs. A246^IC^ *p* = 0.1087) (Figure 1E).

Negative current injections elicited hyperpolarizing membrane responses (sags) consistent with the activation of hyperpolarization-gated cyclic nucleotide-activated ion channels (HCN). Quantification of the change in membrane potential (ΔMP) evidenced upon −100 pA current injections revealed smaller hyperpolarizing sags in fAD iNs compared to their isogenic controls (S290C vs. S290^IC^ *p* = 0.016257; A246E vs. A246^IC^ *p* = 0.015170) (Figure 1F).

The rheobase (the minimum current injection required to induce one action potential, AP) was higher in both fAD iNs (S290C: 41.5 ± 9.8 pA, *n* = 13; A246E: 66.3 ± 9.8 pA, *n* = 8) compared to their corrected controls S290^IC^ (19.5 ± 3.0 pA, *n* = 21; *p* < 0.05) and A246^IC^ (22.2 ± 4.7 pA, *n* = 10; *p* < 0.01), respectively (Figure 1G). Notably, positive current injections (up to 140 pA) elicited AP firing in all iNs, but multiple action potentials were only observed in corrected iNs (Figure 1B). Accordingly, the number of APs fired upon current injections equivalent to two times the rheobase (#AP@2xRheo) was significantly lower in both fAD lines compared to corrected iNs (Figure 1H). Thus, iNs derived from *PSEN1* S290C iPSCs fired 2.0 ± 0.7 action potentials (*n* = 12) compared to 6.6 ± 0.8 (*n* = 21) APs by S290^IC^ (*p* < 0.001), and *PSEN1* A246E fired 1.4 ± 0.4 APs (*n* = 8) compared to 4.0 ± 1.0 APs (*n* = 9) by A246^IC^ iNs (*p* < 0.05). Thus, neurons derived from fAD patients with S290C or A246E mutations in *PSEN1* exhibited reduced firing capacity and excitability compared to their isogenic controls, indicating a common phenotype of reduced neuronal excitability.

### 3.2 iNs from different fAD *PSEN1* backgrounds display distinct AP characteristics

**Figure 2:**
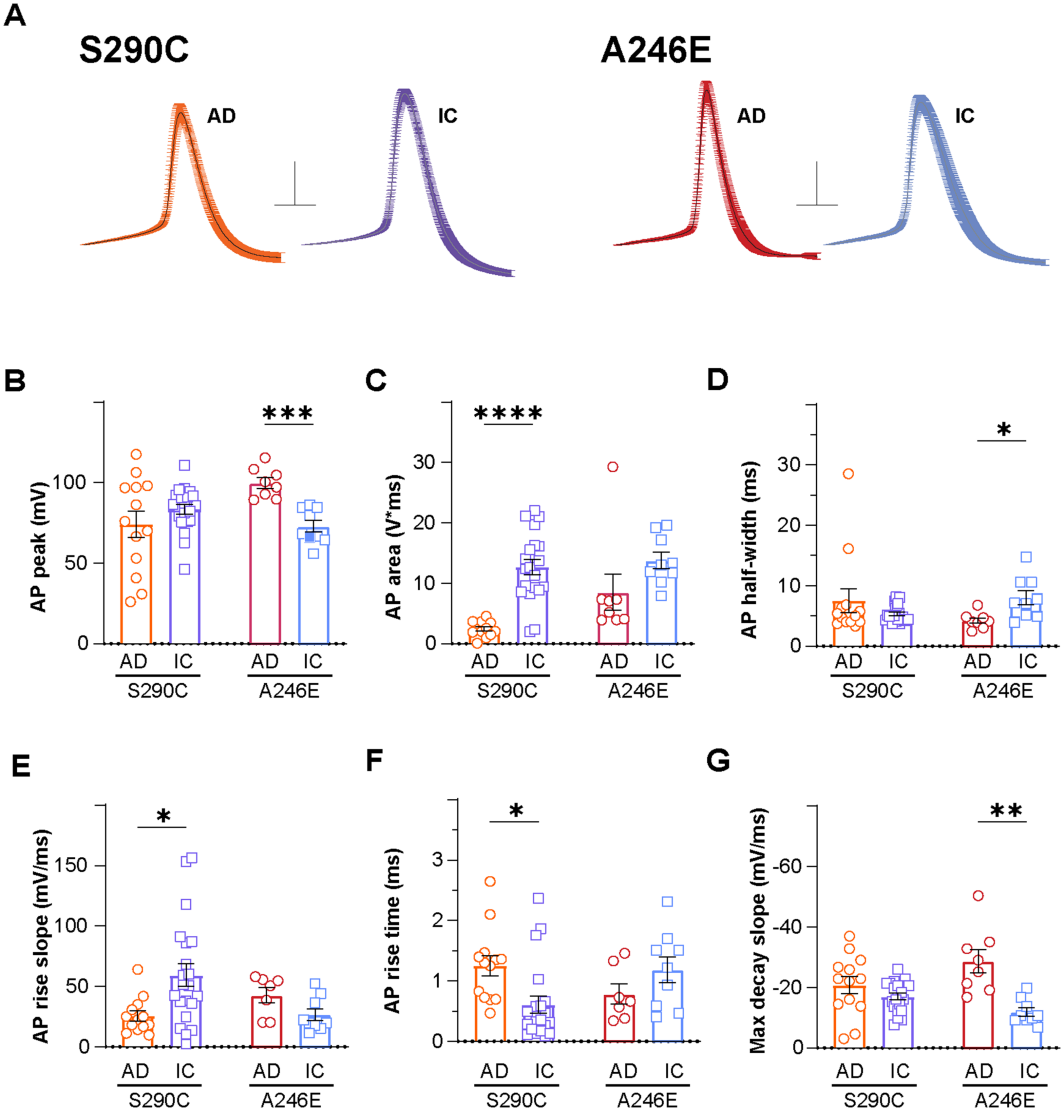
AD induced neurons display altered action potential properties compared to isogenic controls. Action potential properties were also compared between AD patient lines with *PSEN1* mutations (S290C and A246E) and isogenic controls, including **(A)** action potential waveforms, where the first action potential from each neuron fired at rheobase was aligned at the point of initiation and the resulting mean ± SEM waveforms are presented (Scale: 20 mV, 5 ms), **(B)** peak amplitude, **(C)** area, **(D)** half-width, **(E)** rise slope, **(F)** rise time and **(G)** max decay slope. Data is presented as the mean ± SEM. Each data point represents an individual cell (*n* = 7–41), from 3 independent differentiations. Data was analyzed using multiple t-tests with Holm-Sidak for multiple comparisons where *p < 0.05, **p < 0.01, ***p < 0.001, and ****p < 0.0001. AD, Alzheimer’s disease; IC, isogenic control.

Given the observed inter-group variability in AP waveforms, the first action potentials fired by iNs from each group were aligned at the point of initiation and averaged for illustration purposes (Figure 2A). Comparative analysis of AP shapes (at rheobase) between fAD and corrected iNs unveiled disparities in AP peak amplitude, area, half-width, rise time, rise slope, and maximal decay slope, as outlined in Supplementary Table S1. While AP peak amplitude showed no variance between S290C and S290^IC^ neurons (Figure 2B), their AP area differed significantly, with S290C iNs exhibiting over a 5-fold smaller area than their isogenic control (2229.0 ± 423.5 mV*ms, *n* = 13; vs. 12036.2 ± 1390.3 mV*ms, *n* = 21; *p* < 0.001) (Figure 2C). Additionally, the AP rise slope was markedly higher in S290^IC^-derived neurons (59.3 ± 9.5 mV/ms, *n* = 21), compared to those harboring the S290C mutation (25.6 ± 4.1 mV/ms, *n* = 13, *p* < 0.05) (Figure 2E). Consequently, the AP rise time was more than 2-fold slower in S290C neurons compared to S290C^IC^ (1.3 ± 0.2 ms, *n* = 13 vs. 0.6 ± 0.2 ms, *n* = 20, *p* < 0.05) (Figure 2F) with no discernible differences in AP maximal decay slope (*p* = 0.15) (Figure 2G).

In contrast, PSEN1 A246E fAD iNs exhibited higher AP peak amplitudes (99.7 ± 3.3 mV, *n* = 8) compared to corrected A246^IC^ iNs (72.9 ± 3.7 mV, *n* = 9; *p* < 0.001) (Figure 2B), while showing no significant difference in AP area (*p* = 0.44) (Figure 2C). Despite similar AP rise parameters (Figures 1C,E,F), A246E neurons displayed narrower APs than their isogenic control (AP half-width 4.2 ± 0.5 ms, *n* = 8 vs. 8.0 ± 1.2 ms, *n* = 9, respectively; *p* < 0.05) (Figure 2D), accompanied by markedly larger maximal decay slopes (−28.7 ± 3.8 mV/ms, *n* = 8 vs. −11.9 ± 1.4 mV/ms, *n* = 9, *p* < 0.01) (Figure 2G). Thus, the action potential shape in fAD iNs appears distinctively affected by specific *PSEN1* mutations.

### 3.3 Voltage-gated potassium currents are larger in iNs from both *PSEN1* fAD patients

**Figure 3:**
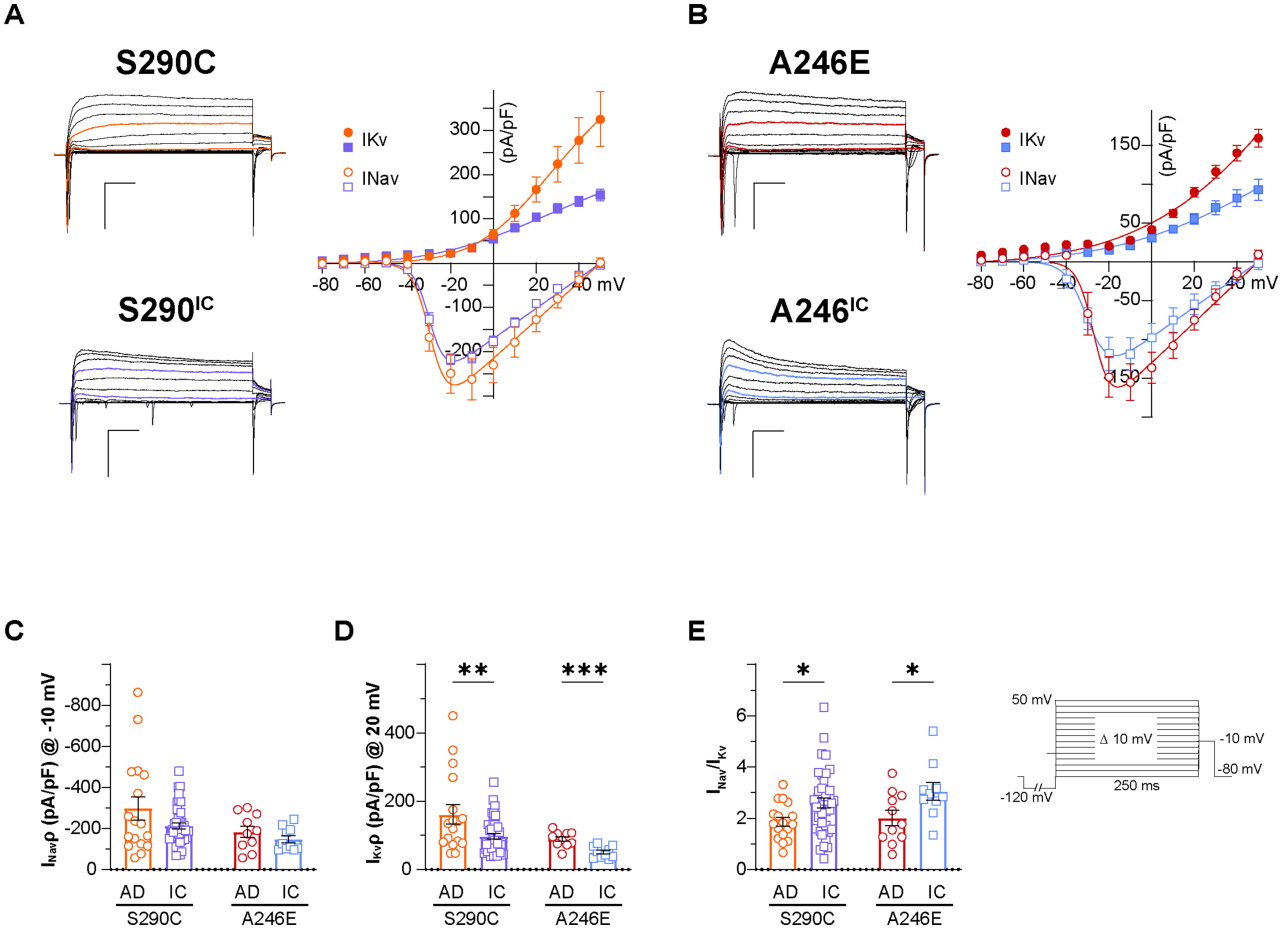
Voltage-dependent conductances in neurons derived from AD patients with a *PSEN1* mutation and their isogenic controls. Whole cell patch-clamp recordings were performed on day 21–35 NGN2 iNs derived from S290C (PSEN1 S290C) and A246E (PSEN1 A246E) and their respective CRISPR corrected controls, S290^IC^ and A246^IC^ cell lines. **(A)** Left: representative traces of voltage-dependent currents elicited by 250 ms square pulses from −80 mV to 50 mV in 10 mV steps (Vh −80 mV, 0.1 Hz) in S290C (orange) and S290^IC^ (purple) neurons. A 250 ms pre-pulse to −120 mV was used to minimize cumulative inactivation (inset). Right: average I–V relationships of peak inward (INav) and outward current (IKv). Data is presented ± SEM (*n* = 35). **(B)** Left: representative traces of voltage-dependent currents elicited by 250 ms square pulses from −80 mV to 50 mV in 10 mV steps (Vh −80 mV, 0.1 Hz) in A246E (red) and A246^IC^ (blue) neurons. A 250 ms pre-pulse to −120 mV was used to minimize cumulative inactivation (inset). Right: average I–V relationships of peak inward (INav) and outward current (IKv). Data is presented ± SEM (*n* = 35). **(C)** Nav and **(D)** Kv current density quantification. **(E)** Nav to Kv current ratio. Data is presented as the mean ± SEM. Each data point represents an individual cell (*n* = 10–41), from 3 independent differentiations. Data was analyzed using multiple t-tests with Holm-Sidak for multiple comparisons where *p < 0.05, **p < 0.01, ***p < 0.001, and ****p < 0.0001. AD, Alzheimer’s disease; IC, isogenic control.

The shape of the action potential is intricately governed by factors, such as ion channel dynamics and the equilibrium between inward and outward currents, primarily mediated by voltage-gated sodium (Nav) and potassium (Kv) channels, respectively. Representative iN whole-cell currents elicited by 250 ms step depolarization (from −80 mV to 50 mV; Vh −80 mV, inset) recorded in voltage-clamp mode are displayed for S290C and S290IC (Figure 3A) and A246E and A246IC neurons (Figure 3B). To assess the ability of iNs to initiate and terminate action potential firing, Nav and Kv currents were analyzed. Nav channels, which drive rapid depolarization, open quickly near the threshold for action potential initiation (~ −40 to −20 mV). Sodium currents were measured at −10 mV to ensure the activation of a large fraction of available Nav channels. Kv channels, responsible for restoring the resting membrane potential and regulating action potential duration and firing frequency, activate more slowly at more depolarized potentials (~ −10 to +20 mV). Kv currents were therefore quantified at 20 mV to capture maximal outward potassium flow. Total Nav and Kv currents (at −10 mV and 20 mV, respectively) were normalized to the cell capacitance and expressed as current density (pA/pF). The disease lines and isogenic corrected controls demonstrated similar AP thresholds (Supplementary Table S2), indicating no significant differences in the voltage required to trigger an action potential. In alignment with these findings, no detectable differences in Nav channel-mediated currents were observed between these groups (Figure 3C). In contrast, Kv channel mediated currents were significantly larger in neurons derived from patients with fAD *PSEN1* mutations (S290C: 161.5 ± 28.1 pA/pF, *n* = 17; A246E: 89.2 ± 7.1 pA/pF, *n* = 10), compared to their respective CRISPR-corrected isogenic controls (S290^IC^: 97.0 ± 8.1 pA/pF, *n* = 41; *p* < 0.01; A246^IC^: 50.7 ± 5.4, *n* = 10; *p* < 0.001) (Figure 3D). Consequently, the ratio between Nav and Kv current densities (INav/IKv) was significantly larger for both isogenic controls, compared to their respective fAD lines (*p* < 0.05) (Figure 3E). Similarly, the ratio of maximal Nav and Kv conductances (from conductance-voltage relationships) was higher in the corrected lines (Supplementary Table S2), suggesting a potential imbalance between inward and outward currents as a possible cause of reduced excitability in *PSEN1* fAD iNs.

## 4 Discussion

This study investigated early excitability changes in fAD using iPSC-derived neurons from patients bearing *PSEN1* mutations. These neurons showed reduced firing responses suggestive of altered ionic homeostasis. Action potential properties in neurons bearing S290C or A246E mutations diverged, indicating varied disrupted pathways, however the overall impact on firing was consistent amongst the mutations. Understanding these early cellular and molecular changes may shed light on cause and progression in fAD.

There have been over 300 mutations in the *PSEN1* gene identified with varying pathogenicity and effects on neuropathology and molecular pathways. This study has provided evidence of common intrinsic excitability changes that are altered in neurons with a *PSEN1* S290C or A246E mutations. The S290C mutation occurs due to a missense mutation in the splice acceptor site on the boundary of intron 8 and exon 9, resulting in the skipping of exon 9, which leads to impaired APP cleavage and Aβ processing (Rovelet-Lecrux et al., 2015; Steiner et al., 1999). The deletion of exon 9 impairs cellular functions including calcium influx in hippocampal neurons (Skobeleva et al., 2022), altered mitochondrial metabolism, calcium homeostasis and inflammation in astrocytes (Oksanen et al., 2017), impaired endocytosis in neurons (Woodruff et al., 2016) and lipid metabolism (Landman et al., 2006). In this research, S290C iNs exhibited reduced capacitance, supported by reduced neurite length and branch points, and a decreased Nav/Kv current density ratio, likely underscoring the smaller AP area/rise parameters and overall impaired neuronal excitability. The A246E mutation is also pathogenic and occurs in exon 7 of *PSEN1* (Sherrington et al., 1995), impeding proper APP cleavage and resulting in toxic amyloid peptide generation (Mahairaki et al., 2014; Yang et al., 2017). In a cellular context the A246E mutation results in impaired microglial differentiation (Aubert et al., 2023), premature neuronal differentiation (Yang et al., 2017), altered astrocyte metabolic function and inflammatory activation (Elsworthy et al., 2023) and increased neuronal susceptibility to Aβ (Armijo et al., 2017). In this study, iNs derived from patients with *PSEN1* mutations showed decreased hyperpolarizing responses which together with an Nav/Kv imbalance would require stronger current injection (rheobase) for AP firing.

The observed reduced neuronal excitability in iNs in this study is consistent with previous findings on *PSEN1* fAD mutations. For instance, cortical organoids with an L345F mutation displayed reduced extracellular network activity measured by multi-electrode array analysis compared to its isogenic control, likely due to altered notch signaling (Hurley et al., 2023). The A246E cell line used in this study, was demonstrated to have a deficiency in Notch1 in iNs, resulting in susceptibility to ferroptosis (Greenough et al., 2022). Future work will evaluate the differentiation and maturation capacity of A246E neurons. In APP_SWE_/PSEN1(dE9) transgenic mice, hippocampal neurons exhibited increased rheobase, decreased action potential frequency at lower current injections (30 and 50 pA), and increased frequency at 100 pA (Chen et al., 2021). Time-dependent changes in spike frequency of CA1 pyramidal neurons were observed in the APP_SWE_/PSEN1(dE9) transgenic mouse model, with reduced spike counts compared to wild-type at 1 month of age, no differences at 4 months and increased spikes at 10 months, suggesting excitability is altered with disease progression (Vitale et al., 2021). In an amyotrophic lateral sclerosis mouse model, it is the neurons that lose the ability to fire repetitively that become vulnerable and are lost as the disease progresses (Buskila et al., 2019). We hypothesize that *PSEN1* mutations make neurons more susceptible to excitability changes, even in the absence of overt Aβ and tau pathology, which may contribute to neuronal loss in disease.

Future studies should investigate the impact of the S290C mutation on Notch signaling and neuronal differentiation, as many mutations have been linked to impaired cleavage of Notch1, resulting in impaired neurogenesis or premature differentiation, which may contribute to the altered excitability changes. Zhang et al. (2020) showed the *PSEN1* S169 deletion mutation, induced AD pathology and cognitive deficits in a Notch signaling independent pathway, suggesting different *PSEN1* mutations may contribute to neuronal deficits via distinct mechanisms. This phenomenon, in addition to different ion channels being dysregulated, may also explain specific impacts on action potential properties between lines. Common to both mutations was enhanced Kv conductance, likely implicated in hypoexcitability. Kv1 channels are known to regulate the repression of intrinsic excitability and synaptic transmission (Colasante et al., 2020; Thouta et al., 2021) and are upregulated in AD proteomic datasets (Askenazi et al., 2023). Nevertheless, the vast collection of potassium channels expressed in neurons and glia warrant future work identifying Kv channel expression changes in fAD *PSEN1* mutant cell lines, as well as investigating the effects of introducing the same *PSEN1* mutations into otherwise healthy cell lines to determine direct causal links between presenilin-1 dysfunction and Kv channel dysregulation.

In a reductionist model, this study revealed a common hypoexcitability phenotype in iNs generated from fAD patients with *PSEN1* mutations in the absence of Aβ and tau disease pathology (Targa Dias Anastacio et al., 2024) and supporting cell types. Future research may employ genome editing to create cell lines with *PSEN1* mutations of varying pathogenicity to establish a potential link between neuronal excitability and early onset-AD progression. In animal models, Aß appears to induce neuronal hyperexcitability, while soluble mutant tau suppresses neural activity in the rTg4510 and P301S tau transgenic mouse models (Busche et al., 2019; Marinković et al., 2019; Menkes-Caspi et al., 2015). Ghatak et al. (2019) reported hyperexcitability in co-cultures of astrocytes and iPSC-derived neurons from fAD patients. This yields an interesting observation, since astrocytes and other glial cells regulate excitability in AD (Targa Dias Anastacio et al., 2022), whereas in isolation, the excitatory neurons demonstrate higher current density of voltage-gated potassium channels. It is hypothesized that early stages of AD results in Aß-dependent hyperexcitability, preceding the onset of tau-dependent hypoactive neural circuits (Harris et al., 2020). Differences in neuronal maturity, Aß and tau generation between cell lines and co-culture may influence neural circuits *in vitro*. To determine the interplay between *PSEN1* mutations, AD pathology and neuronal excitability, simplistic 2D models may not be sufficient and would require more advanced systems such as 3D cerebral organoids matured long-term. It is also important to consider how support cells, such as oligodendrocytes, astrocytes and microglia, influence the disruption to neuronal activity and transition between hypo- and hyperexcitability.

In summary, this study demonstrates that neurons derived from AD patients with *PSEN1* mutations exhibit reduced firing activity and altered electrophysiological properties. Mechanistic understanding of the early changes disrupted in AD will provide insight into the driving forces of neurodegeneration and provide novel avenues for intervention to slow this devastating disease.

## Data Availability

The raw data supporting the conclusions of this article will be made available by the authors, without undue reservation.

## Footnotes

^1^ https://www.alzforum.org/mutations/psen-1
